# Disturbance Promotes Non-Indigenous Bacterial Invasion in Soil Microcosms: Analysis of the Roles of Resource Availability and Community Structure

**DOI:** 10.1371/journal.pone.0045306

**Published:** 2012-10-02

**Authors:** Manqiang Liu, Lisa Bjørnlund, Regin Rønn, Søren Christensen, Flemming Ekelund

**Affiliations:** 1 Soil Ecology Lab, College of Resources and Environmental Sciences, Nanjing Agricultural University, Nanjing, China; 2 Terrestrial Ecology Section, Department of Biology, University of Copenhagen, Copenhagen K, Denmark; U. S. Salinity Lab, United States of America

## Abstract

**Background:**

Invasion-biology is largely based on non-experimental observation of larger organisms. Here, we apply an experimental approach to the subject. By using microbial-based microcosm-experiments, invasion-biology can be placed on firmer experimental, and hence, less anecdotal ground. A better understanding of the mechanisms that govern invasion-success of bacteria in soil communities will provide knowledge on the factors that hinder successful establishment of bacteria artificially inoculated into soil, e.g. for remediation purposes. Further, it will yield valuable information on general principles of invasion biology in other domains of life.

**Methodology/Principal Findings:**

Here, we studied invasion and establishment success of GFP-tagged *Pseudomonas fluorescens* DSM 50090 in laboratory microcosms during a 42-day period. We used soil heating to create a disturbance gradient, and hypothesized that increased disturbance would facilitate invasion; our experiments confirmed this hypothesis. We suggest that the key factors associated with the heating disturbance that explain the enhanced invasion success are increased carbon substrate availability and reduced diversity, and thus, competition- and predation-release. In a second experiment we therefore separated the effects of increased carbon availability and decreased diversity. Here, we demonstrated that the effect of the indigenous soil community on bacterial invasion was stronger than that of resource availability. In particular, introduced bacteria established better in a long term perspective at lower diversity and predation pressure.

**Conclusion:**

We propose increased use of microbial systems, for experimental study of invasion scenarios. They offer a simple and cost-efficient way to study and understand biological invasion. Consequently such systems can help us to better predict the mechanisms controlling changes in stability of communities and ecosystems. This is becoming increasingly relevant since anthropogenic disturbance causes increasing global change, which promotes invasion. Moreover, a thorough understanding of factors controlling invasion and establishment of artificially amended micro-organisms will mean a major step forward for soil-remediation microbiology.

## Introduction

The concept of biological invasion plays an essential role in the study of biodiversity maintenance and ecosystem services [1]–[3]. Thus, a better understanding of the factors that control invasion can assist in the management of habitats, if the susceptibility to unwanted species can be regulated. Current hypotheses on mechanisms behind biological invasions mainly stem from macro-ecology, which have emphasized the importance of disturbance [1], species diversity [4], community composition [5], and resource availability [6]. Hence, invasion theory is largely based on observation and not on experiments. Disturbance is recognized as one of the most important factors to promote biological invasions because it can, concomitantly, alter community composition, diversity, as well as resource availability [6]–[9]. This has been demonstrated by several empirical studies [5], [6], [10], [11].

Hence, experimental disturbance studies provide a way to explore the integrated effects of resource and community changes. Most studies on impacts of disturbance on ecosystem stability, including community resistance to invasions, have focused exclusively on macro-organisms [12]. Lately, however, the stability of soil microbial communities and their functioning to disturbance has attracted more attention [8], [13]. Invasion is considered a serious environmental issue in macro-ecology. Oppositely, the failure of invasion and establishment of artificially amended micro-organisms (e.g. for soil-remediation purposes) is considered a serious issue in applied microbial ecology. Besides, microbial communities in soil are strongly related to above-ground functioning including invasion of plants [14]. Still, only few studies have explored the effects of disturbance on microbial invasion [12].

Disturbance can directly increase soil organic matter availability by exposing new substrate by breaking soil aggregates and by killing members of the native community [15], [16]. Further it can affect soil community diversity and structure [16]–[18]. Van Elsas *et al* [11] manipulated soil community complexity via progressively increasing disturbance intensity using fumigation. They introduced non-indigenous bacteria 60 days after disturbance to avoid confounding effects of temporary resource enrichment; hence they also excluded the possibility to see invasion success in a strongly disturbed community.

The use of soil microcosms offers a simple, fast and cost-efficient approach to perform experimental studies of the mechanisms behind biological invasions. To our knowledge, no study has previously explored the concomitant contributions of community diversity changes, resource availability and their interaction on invasion in microbial systems. Therefore, we conducted two sequential microcosm-experiments where we investigated the effect of disturbance exemplified by heating on the ability of a bacterium (*Pseudomonas fluorescens* DSM 50090) to colonize and establish in soil. In experiment 1, we simply examined the effect of heating. In experiment 2 we separated the effects of carbon release and community disturbance. We hypothesized that: 1) Microbial invasion success will increase with disturbance intensity, due to the combined effects of resource enrichment and recipient community disturbance and, 2) The relative importance of community disturbance compared to resource enrichment will increase with time following disturbance, as ephemeral resources are depleted, and the recipient system recovers. We found experimental evidence that confirmed both hypotheses.

## Results

### First experiment: effect of heating

**Figure 1:**
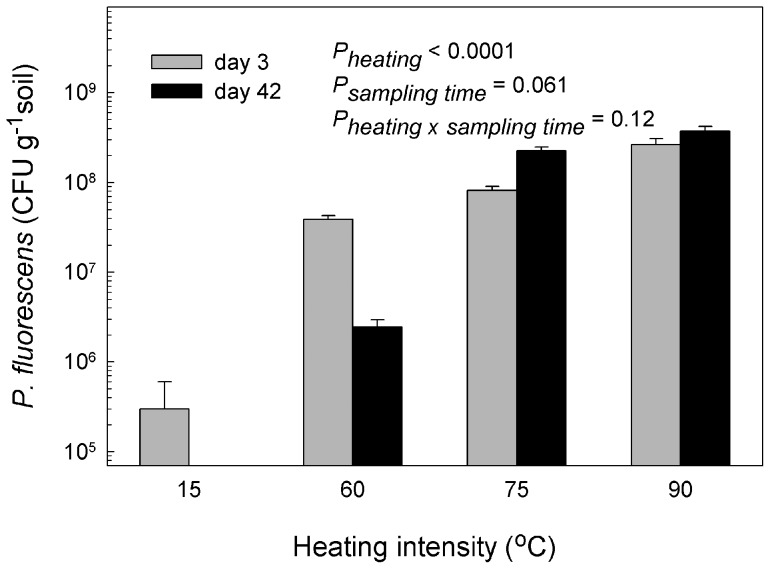
Heating facilitated invasion of *Pseudomonas fluorescens* DSM 50090. Soil microcosms were exposed to four different heating intensities (15°C, 60°C, 75°C or 90°C) for 24 h and subsequently amended with *P. fluorescens* DSM 50090. Culturable *P. fluorescens* were recovered from sampling of soil microcosms to reveal its ability to invade (sampling at day 3) and establish (sampling at day 42) in the systems. We used a two-way ANOVA with sampling time and heating intensity as quantitative variables to test the results. Data were log transformed prior to analysis to equalize variances. *P*-values <0.15 are shown.

We exposed soil microcosms to four different heating intensities (15°C, 60°C, 75°C or 90°C) for 24 h. These heating intensities were chosen on the basis of a preliminary experiment including six temperatures (15°C, 30°C, 45°C, 60°C, 75°C and 90°C), which showed that heating to below 60°C had no severe impact on soil biodiversity. Subsequently, we introduced the non-indigenous *Pseudomonas fluorescens* DSM 50090 into the systems. After 3 days the number of *P. fluorescens* DSM 50090 correlated positively with increasing heating intensity (Figure 1). This trend persisted after 42 days, but whereas *P. fluorescens* DSM 50090 decreased in numbers for the 15 and 60°C heating-treatments from the first to the second sampling, in the 75°C and 90°C treatments, *P. fluorescens* DSM 50090 increased by 178% and 28%, respectively, between the two samplings.

**Figure 2:**
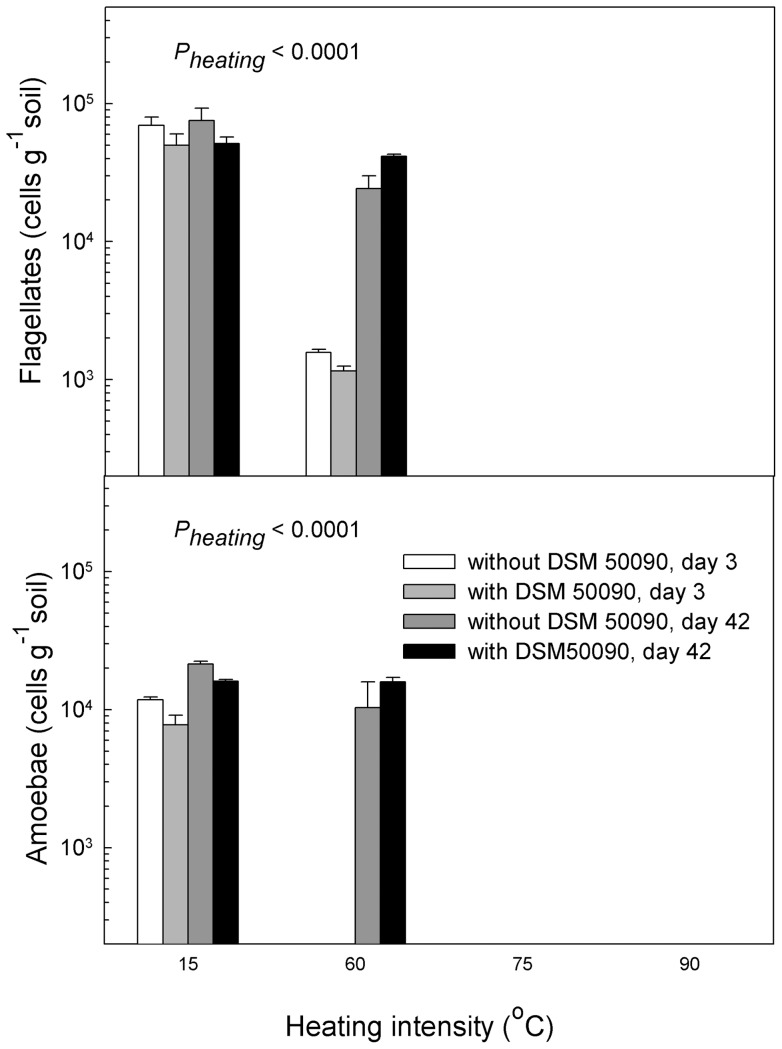
Heating decreased protozoan numbers. Soil microcosms were exposed to four different heating intensities (15°C, 60°C, 75°C or 90°C) for 24 h and subsequently amended with either *P. fluorescens* DSM 50090 or just with phosphate buffer. Culturable protozoa (heterotrophic flagellates: upper panel, naked amoebae: lower panel) were measured after 3 and 42 days. Heating above 60°C completely eliminated the protozoa, whereas amendment with *P. fluorescens* had no effect. We used a three-way ANOVA with sampling time and heating intensity as quantitative variables and *Pseudomonas fluorescens* DSM 50090 as qualitative variable to test the results. Data were log transformed prior to analysis to equalize variances. P-values <0.05 are shown.

In all heated treatments (>15°C) the numbers of protozoa (flagellates and amoebae) were strongly reduced at the first sampling (Figure 2). In the 60°C-microcosms, protozoa recovered to a great extent at the second sampling, whereas the 75°C and 90°C treatments remained completely devoid of protozoa.

**Figure 3:**
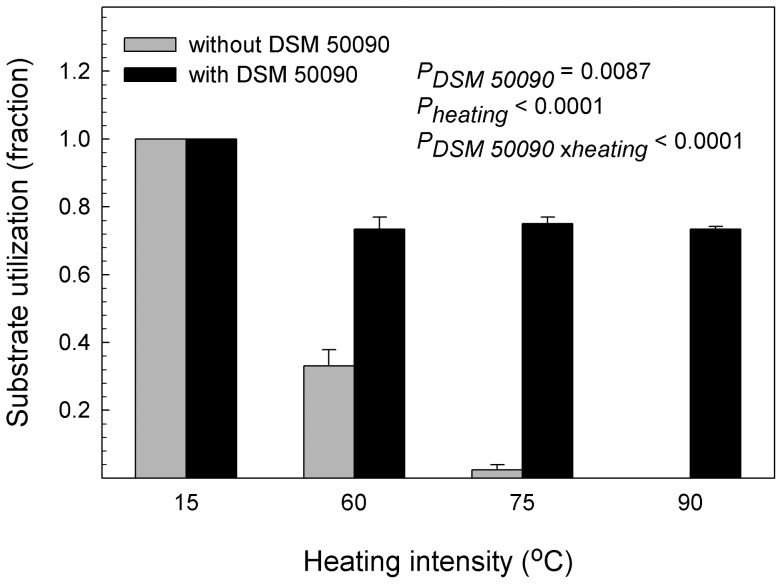
Microbial functional diversity decreased with heating; *P. fluorescens* alone provides 75 % of the diversity. Soil microcosms were exposed to four different heating intensities (15°C, 60°C, 75°C or 90°C) for 24 h and subsequently amended with either *P. fluorescens* DSM 50090 or just with phosphate buffer and incubated for 42 days. Microbial diversity was measured as the fraction of substrates metabolised in Biolog EcoPlates with soil from the microcosms. We used a two-way ANOVA with heating intensity as quantitative variables and *P. fluorescens* as qualitative variable to test the results., data were Arcsine-square root transformed to equalize variances. *P*-values <0.05 are shown.

Functional diversity of indigenous micro-organisms, measured on day 42 as fraction of positive wells in Biolog EcoPlates (Figure 3), declined sharply with increasing heating intensity. In the 15°C-treatment, all substrate types in the EcoPlates were utilized, followed by 33%, 2% and 0% for the 60°C, 75°C and 90°C treatments, respectively. Surprisingly, amendment with *P. fluorescens* DSM 50090 alone could provide 75 % of the functional diversity in the Biolog EcoPlates.

Indigenous CFU-numbers also declined with increasing heating intensity, in systems without *P. fluorescens* DSM 50090. At the first sampling, only 52% and 32% of the number in the 15°C-treatment (2.2×10^7^ CFUs g^−1^) were left at 60°C and 75°C, respectively (Data not shown). At the second sampling, indigenous bacterial CFU numbers in the 60°C treatment made up 31% of the 15°C-treatment (Data not shown). No indigenous bacteria were detected in the 90°C microcosms at any of the samplings.

### Second experiment: separation of carbon and community change effects

In the second experiment, we separated the heat treatment effects on community disturbance and carbon release. We did so by leaching the microcosms after heating as to remove soluble carbon. Subsequently, we then amended all leached heating treatments with carbon amounts corresponding to amounts produced at the four heating treatments. Amendment with *P. fluorescens* DSM 50090, incubation, CO_2_ measurement and destructive sampling were as in Experiment I.

**Figure 4:**
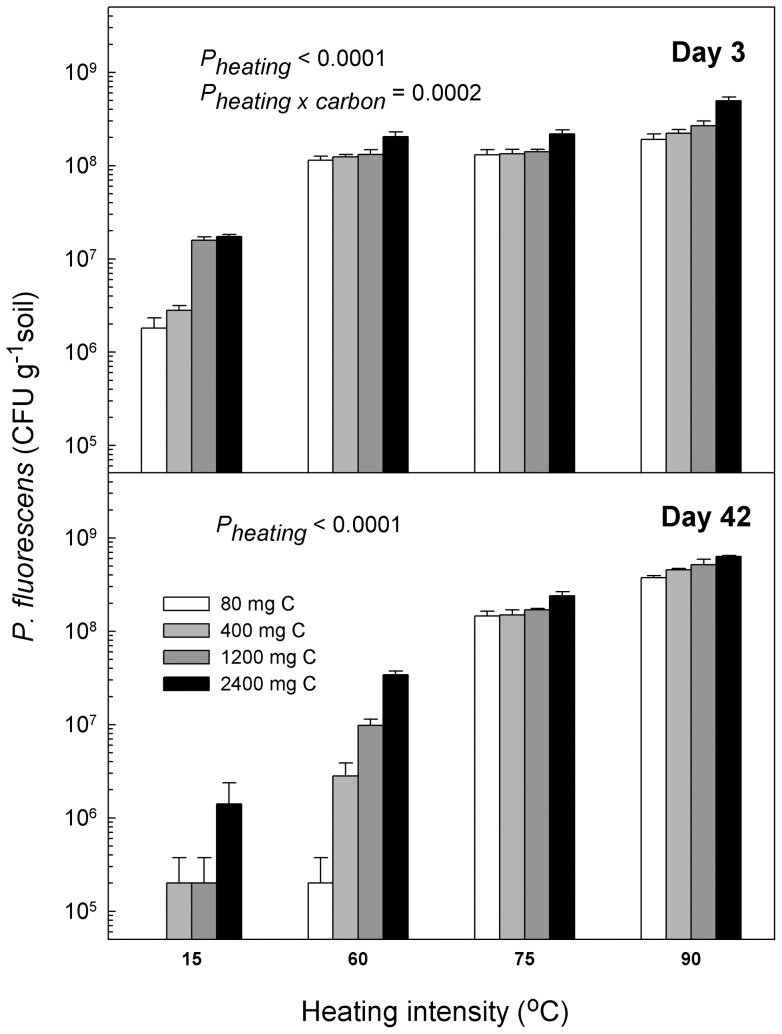
Heating facilitates invasion of *Pseudomonas fluorescens* DSM 50090. We studied invasion and establishment of the bacterium *P. fluorescens* DSM 50090 in soil microcosms exposed to different heating intensities (15°C, 60°C, 75°C or 90°C) for 24 h and subsequently amended with the bacterium. After heating we washed the microcosms to remove soluble carbon, and to each heating treatment we subsequently amended soluble carbon corresponding to the amount produced from the four heating treatments to separate the effects of heating on community structure and on release of soluble carbon. Heating permanently facilitated *P*. *fluorescens* DSM 50090, whereas the effect of carbon was transient. We performed two separate two-way ANOVA's (heating intensity *×* carbon amendment) for the two samplings. P-values refer to these two-way ANOVA's performed on the two different depicted situations. Data were log transformed to equalize variances.

A three-way analysis of variance (ANOVA) (heating × sampling time × carbon amendment) revealed that heating significantly facilitated invasion of *P. fluorescens* DSM 50090 (*P_heating_*  =  0.03), whereas the effect of carbon amendment depended on sampling time (*P_carbon × sampling time_*  =  0.046). To analyze this further, we separated the data sets for day 3 and day 42 and performed two separate two-way ANOVAs (heating × carbon amendment) for the two samplings (Figure 4). Here we found that heating significantly increased abundance of *P. fluorescens* DSM 50090 at both samplings (*P_heating_* <0.0001). But whereas, 0n day 3, carbon amendment amplified the effect of heating significantly (*P_carbon × heating_*  = 0.0002), this effect was not significant at day 42, although (*P_carbon × heating_*  = 0.15). Thus, carbon amendment affected *P. fluorescens* DSM 50090 much stronger at day 3 than at day 42 (Figure 4).

**Figure 5:**
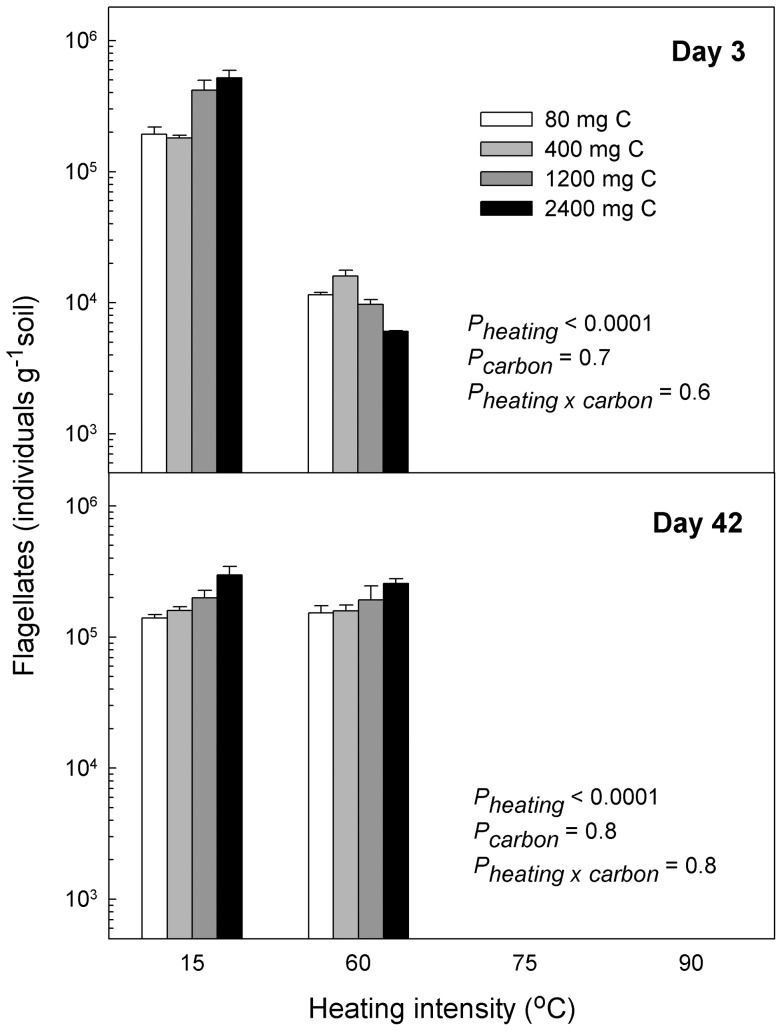
Heating decreased flagellate numbers. We studied survival of cultivable heterotrophic flagellates in soil microcosms exposed to different heating intensities (15°C, 60°C, 75°C or 90°C) for 24 h. After heating we washed the microcosms to remove soluble carbon, and to each heating treatment we subsequently amended soluble carbon corresponding to that produced from the four heating treatments to separate the effects of heating on community structure and on release of soluble carbon. Heating to 75°C or 90°C permanently eliminated heterotrophic flagellates, whereas they recovered at 60°C heating. Carbon amendment had no significant effect on the heterotrophic flagellates. *P*-values refer to two-way ANOVA's performed on the two different situations: data were log transformed to equalize variances; all *P*-values shown.

**Figure 6:**
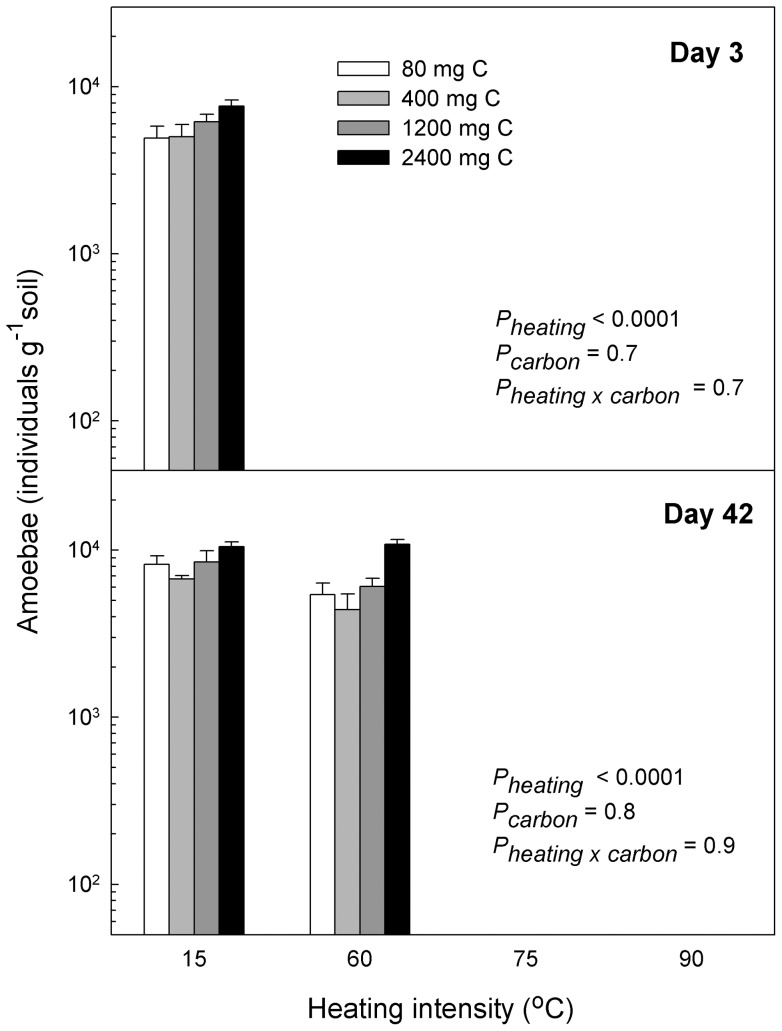
Heating decreased numbers of amoebae. We studied survival of cultivable amoebae in soil microcosms exposed to different heating intensities (15°C, 60°C, 75°C or 90°C) for 24 h. After heating we washed the microcosms to remove soluble carbon, and to each heating treatment we subsequently amended soluble carbon corresponding to that produced from the four heating treatments to separate the effects of heating on community structure and on release of soluble carbon. Heating to 75°C or 90°C permanently eliminated amoebae whereas they recovered at 60°C heating. Carbon amendment had no significant effect on the amoebae. *P*-values refer to two-way ANOVA's performed on the two different situations: data were log transformed to equalize variances; all *P*-values shown.

Protozoan numbers were only slightly affected by carbon amendment, though the treatments with highest carbon amendment generally had the highest numbers of flagellates and amoebae (Figures 5, 6). As in Experiment I, numbers of protozoa (especially amoebae) in the 60°C-heating treatment increased strongly between samplings (Figures 5, 6).

## Discussion

### First experiment: Disturbance facilitates colonization

As we anticipated, increased heating intensity caused increased system disturbance; indicated by reduced bacterial and protozoan numbers and by reduced functional diversity. In line with our hypotheses, we also observed that heat treatment facilitated invasion by *P. fluorescens* DSM 50090. Elton [4] hypothesized that high biodiversity impeded invasive organisms; a view which is shared by [11] and [19] for microbial communities. The decreased functional diversity at increased disturbance agrees with the conception that disturbance may reduce competition by decreasing indigenous diversity and biomass [20]. Predation is another factor, which impedes intrusion of foreign bacteria [21]; in line with this, we observed that protozoan numbers were inversely related to successful invasion of *P. fluorescens* DSM 50090.

The easily decomposable carbon released during heating may likewise facilitate invasion of *P. fluorescens* DSM 50090. Community resistance may be more important than avoiding initial invasion in reducing establishment of invaders [22]. Thus, we suggest that the combination of resource limitation and the successional recovery of the disturbed community towards a more mature stage will result in increasing relative importance of biotic resistance. We observed that under low or intermediate disturbance (i.e. 15°C and 60°C heating), *P. fluorescens* DSM 50090 declined from day 3 to day 42. In the early successional stages, the fast growing *P. fluorescens* DSM 50090 will benefit from both carbon produced during heating and from low competition and predation [23]. However, with time, resources will be depleted concomitantly with recovery of predators and competitors. At the more intense heating, (i.e. 75°C and 90°C), where the indigenous populations of competitors and predators are permanently reduced, *P. fluorescens* DSM 50090 can persist. To further explore the situation, we separated the heating effects on the indigenous community from the heating effects on the carbon release in the second experiment.

### Second experiment: Separation of resource amendment and diversity reduction effects

We observed that resource amendment as well as community-diversity-reduction facilitated invasion of *P. fluorescens* DSM 50090 in our microcosms. Recently, the relative importance of resource availability and community diversity in determining the susceptibility of communities to invasion has become an issue. It has been suggested that resource availability is a primary factor in determining susceptibility to invasion [6], [24], [25]. Other researchers have though concluded that community diversity played the key role in resistance to invasion regardless of resource availability [26], [27]. In our experiments, diversity reduction was the more important factor for facilitation of invasion of *P. fluorescens* DSM 50090. Even though enhanced resource availability enhanced invasion success, it could not counterbalance the resistance in the systems with relatively higher community diversity.

### Conclusion

Most studies on the relation between disturbance, resource availability, and community structure on one hand, and biological invasion on the other, have been performed in a macro-ecological context, mostly in plant communities, and only a small effort has been done to understand the importance of these factors in determining the invasion potential in microbial systems [12]. Here, we provided experimental evidence that the factors and mechanisms that govern invasion in microbial systems are much the same as in communities with macro-organisms. Increasing disturbance led to increasing invasion success of *P. fluorescens* DSM 50090, which could be attributed to increased resource availability as well as predation and competition release. Our second experiment suggested that effects on community structure were more essential for invasion success than increased resource availability.

We notice that our study may have practical implications for both micro- and macro-biology. In soil-remediation microbiology, it is a major issue that microorganisms artificially amended to soil mostly cannot survive due to competition and predation. The present results suggest that it is possible to facilitate survival of artificially amended soil organisms by accompanying their introduction with addition of organic substances or with soil fumigation. Extrapolation of the results to a macroscopic context, e.g. a plant community, would suggest that a temporary nutrient stress induces a reversible invasion, whereas a permanent habitat diversity-loss would make inversion irreversible. Hence, it could be hypothesized that habitat fragmentation, which permanently reduces habitat diversity, facilitates invasive species more than a transient fertiliser stress.

We suggest using microbial systems for experimental study of invasion scenarios, firstly they offer a simple and cost-efficient way to study and understand the mechanisms controlling biological invasion and consequently help us to better predict changes in stability of communities and ecosystems in response to anthropogenic disturbance and global change. Secondly, a thorough understanding of factors controlling invasion and establishment of artificially amended micro-organisms will mean a major step forward for soil-remediation microbiology.

## Materials and Methods

### Experimental preparations

We collected a surface silt loam soil (0–15 cm) from permanent grassland (Löddeköpinge, Sweden). The location is not privately-owned or protected in any way. No specific permits were required for the described field studies; the field studies did not involve endangered or protected species. The soil samples were homogenized and sieved (<2 mm) to remove large particles. Due to the high organic content (ca. 8%) in the soil, we mixed soil with quartz sand (1∶2) before use. The water holding capacity of soil-sand mixture was about 40%.

Microcosms were now prepared from 15.0 g (dry weight) of the soil-sand mixture in 117 ml serum bottles, each microcosm was further supplied with 3.0 ml distilled water to obtain a water content of 60% of water holding capacity. Bottles were covered with aluminium foil and incubated at 15°C for eight days to decompose easily available soil carbon. The bottles were then sealed with a rubber septum and heated to 15°C, 30°C, 45°C, 60°C, 75°C and 90°C for 24 h. After heating, bottles were cooled and opened in a fume-hood for 2 h to remove CO_2_ released during heating.

Three bottles from each heating treatment were now amended with 3.0 ml soil inoculum (15 g fresh soil shaken with 150 ml sterile water for 1 h, and three replicate bottles from each heating treatment with 3.0 ml sterile water. These bottles were incubated at 15°C for 4 weeks. Accumulated CO_2_ was measured daily at the beginning to weekly at the end of incubation. We found that in the absence of soil inoculum, CO_2_ emission was only significantly affected in microcosms exposed to 60°C, 75°C and 90°C. Hence, we concluded that soil diversity was not severely affected after heating to 30°C or 45°C. Therefore 15°C, 60°C, 75°C and 90°C were chosen for the final experiments.

### First experiment: effect of heating

Prior to the experiments, a GFP-tagged *Pseudomonas fluorescens* DSM 50090 [28] was grown on Tryptic Soy Broth (0.3 gl^−1^, Bacto^TM^, Becton, Dickinson and Company, New Jersey, USA) until mid-exponential growth phase (24 h), washed and re-suspended in phosphate buffer (Modified Neff's Amoeba Saline [29]), and centrifuged (5000 g, 10 min) three times. Microcosms were now heat treated (15°C, 60°C, 75°C, 90°C) and aerated for 2 h as above. For each of the four heating treatments,, we amended eight replicate microcosms with 3.0 ml sterile water containing 10^6^ cells g soil^−1^ GFP-tagged *P. fluorescens* DSM 50090 and eight microcosms with 3.0 ml sterile water without bacteria. Microcosms were incubated at 15°C in the dark. Destructive samplings of four microcosms of each type were conducted twice, i.e. 3 and 42 days after experimental setup.

Sampling was done by adding 60 ml sterile phosphate buffer (Modified Neff's Amoeba Saline, [29]) to each microcosm and shaking them on an end-to-end shaker (180 rpm) for 30 min. The resulting suspension was used for the following procedures: Culturable bacteria were scored on agar-plates as in [30]. Fluorescent colonies of *P. fluorescens* DSM 50090 were counted on a UV transilluminator in a dark room. Protozoa were enumerated as in [31] using a 0.1 gl^−1^ Tryptic Soy Broth medium. Functional diversity was measured in EcoPlates (Biolog, California, USA) at the second sampling by further diluting the soil suspensions prepared above to 2.5×10^−2^. Aliquots of 125 μl of this suspension were added to each well in the EcoPlates, and the plates were incubated in the dark at 15°C for 21 days. The EcoPlates were read at 0 h and once every 24 h for 21 days using a Biorad Benchmark Microplate Reader (plates read at 595 nm; Amphotech Ltd., Massachusetts, USA). An absorbance above 0.5 was regarded as positive. The readings for 0 h were subtracted from all subsequent readings to correct for potential colour development due to particles added. The readings for day 21 were used to estimate the functional diversity, defined as the number of carbon sources utilized.

### Second experiment: separation of carbon and community change effects

We obtained water-extractable organic carbon (WEOC) from heat treated microcosms, as prepared above, by adding 45 ml double distilled water and shaking for 30 min on an end to end shaker (120 rpm.). After sedimentation the liquid was centrifuged (5000 g, 10 min) and filtered (0.8 µm filter). The concentration of organic C in the filtrates was determined by dry combustion using an automated total C analyzer (TOC-5050, Shimadzu Corp., Tokyo, Japan).

To measure biodegradability of WEOC produced by the four heating treatments, we prepared three replicate bottles from 20.0 gram sterile sand, 20.0 ml WEOC from each treatment, and added 1.0 ml soil inoculum suspension (15 g fresh soil shaken with 150 ml water for 1 h, three replicates). Bottles were placed on an end-to end-shaker (150 rpm) at 20°C. CO_2_ accumulated in the bottles were continuously measured until the CO_2_ level was constant after two weeks. We found that the biodegradability of the WEOC for the four temperatures were: 15°C: 29 (1.9)%, 60°C: 32 (2.0)%, 75°C: 53 (2.0)%, and 90°C: 55 (2.6)%; numbers in brackets are standard errors. We only used WEOC obtained from 90°C treatment in the following experiment. By doing this, we minimised the risk of contamination of microcosms from WEOC, as most organisms in the 90°C treatment were killed.

To produce microcosms depleted of WEOC, we added 60 ml sterile water to bottles with soil exposed to each of the four heating levels, shook them (180 rpm, 30 min), centrifuged (5000 g, 10 min) and discarded the supernatant, this procedure was repeated twice. The third supernatant contained the same low WEOC concentration for all heating treatments. Remaining water was evaporated by leaving all bottles in a fume-hood for 24 h. The resulting WEOC depleted microcosms were slightly lower in CFU number (TSA, 0.3 gl^−1^) and functional diversity (indicated by Biolog EcoPlates) but did not differ significantly from unwashed microcosms in these values.

We now set up a factorial experiment. We used four levels of soil heating; i.e. the WEOC depleted microcosms described above, and four levels of resource carbon enrichment, i.e. WEOC from the 90°C treatment diluted to concentrations corresponding to that released from soil under the four heating treatments (i.e. 80, 400, 1200 and 2400 mg C kg^−1^ soil, for the 15°C, 60°C, 75°C and 90°C treatments, respectively). For each of these 16 treatment combinations, we set up six replicate microcosms, all amended with *P. fluorescens* DSM 50090. Amendment with *P. fluorescens* DSM 50090, incubation, CO_2_ measurement, and destructive sampling were as in Experiment I.

### Statistical methods

We used SAS Enterprise Guide 4.1 interface to the SAS 9.1 package (Statistical Analysis System Institute, 2002–2003) to perform two or three way ANOVAs. When needed, data were transformed to equalize variances. We consider *P*-values <0.05 as significant and have thus reported these; to increase clarity larger *P*-values are reported in some cases.
